# Optimising availability and geographical accessibility to emergency obstetric care within a sub-national social health insurance scheme in Nigeria

**DOI:** 10.3389/frhs.2024.1460580

**Published:** 2024-10-16

**Authors:** Aduragbemi Banke-Thomas, Tope Olubodun, Abimbola A. Olaniran, Kerry L. M. Wong, Yash Shah, Daniel C. Achugo, Olakunmi Ogunyemi

**Affiliations:** ^1^ Faculty of Epidemiology and Population Health, London School of Hygiene and Tropical Medicine, London, United Kingdom; ^2^ School of Human Sciences, University of Greenwich, London, United Kingdom; ^3^ Maternal and Reproductive Health Research Collective, Lagos, Nigeria; ^4^ Department of Community Medicine and Primary Care, Federal Medical Centre Abeokuta, Abeokuta, Nigeria; ^5^ Health Systems Strengthening, KIT Royal Tropical Institute, Amsterdam, Netherlands; ^6^ Google Research, Google LLC, Mountain View, CA, United States; ^7^ College of Health Sciences, Nnamdi Azikiwe University, Awka, Nigeria; ^8^ Lagos State Ministry of Health, Ikeja, Nigeria

**Keywords:** universal health coverage, maternal health, emergency obstetric care, geographical accessibility, health insurance

## Abstract

**Introduction:**

Health insurance is a key instrument for a health system on its path to achieving universal health coverage (UHC) and protects individuals from catastrophic health expenditures, especially in health emergencies. However, there are other dimensions to care access beyond financial accessibility. In this study, we assess the geographical accessibility of comprehensive emergency obstetric care (CEmOC) within the Lagos State Health Insurance Scheme.

**Methods:**

We geocoded functional public and private CEmOC facilities, established facilities registered on the insurance panel as of December 2022, and assembled population distribution for women of childbearing age. We used Google Maps Platform's internal directions application programming interface to obtain driving times to facilities. State- and local government area (LGA)-level median travel time (MTT) and a number of CEmOC facilities reachable within 30 min were obtained for peak travel hours.

**Results:**

Across Lagos State, MTT to the nearest public CEmOC was 25 min, reduced to 17 min with private facilities added to the insurance panel. MTT to the nearest public facility in LGAs ranged from 9 min (Lagos Island) to 51 min (Ojo) (median = 25 min). With private facilities added, MTT ranged from 5 min (Agege and Ajeromi-Ifelodun) to 36 min (Ibeju-Lekki) (median = 13 min). On average, no public CEmOC facility was reachable within 30 min of driving for women living in 6 of 20 LGAs. With private facilities included in the scheme, reachable facilities within 30 min remained zero in one LGA (Ibeju-Lekki).

**Conclusions:**

Our innovative approach offers policy-relevant evidence to optimise insurance coverage, support efforts in advancing UHC, ensure coverage for CEmOC, and improve health system performance.

## 1 Introduction

Universal health coverage (UHC) means all people have access to the full range of quality health services needed, when and where they need them, without financial hardship (1). The 2030 Agenda for Sustainable Development emphasises the importance of strengthening comprehensive and coherent methods that ensure that ‘no one is left behind’ in obtaining UHC (2). Health insurance is a key instrument in achieving UHC and may protect people from the financial consequences of out-of-pocket payment (OOP), including catastrophic health expenditure, which individuals may face especially when they suffer health emergencies (1, 3). In many African countries, OOP remains the major source of funding for healthcare (4). However, OOP can be a significant barrier to accessing healthcare and is known to impose a high financial burden on the poor and vulnerable populations (4–6). To forestall the consequences of OOP, African governments at national and sub-national levels have been implementing health insurance schemes (7, 8).

In Nigeria, the most populated country in Africa, the Nigeria National Health Insurance Scheme became operational in 2005 (9, 10), but uptake has remained low, which is estimated at only 3% in 2018 (11). To bridge this coverage gap, several state governments in the country have established sub-national state health insurance schemes, with about 19 states at various stages of implementation (12). In 2015, the Lagos State Government signed the Lagos State Health Scheme (also locally referred to as *Ìlera Èkó*) into law. The scheme had a mandate to protect Lagos residents from catastrophic health expenditure, especially the most vulnerable population. The social health insurance scheme was then officially rolled out in 2020 with a benefit package that covers a range of medical and surgical services including emergency obstetric care (EmOC) (13–15).

EmOC is a package of evidence-based services required to manage potentially life-threatening complications that affect many women during pregnancy, childbirth, and the immediate postpartum period. There are two levels of EmOC service provision: basic and comprehensive. Basic EmOC includes six services, otherwise known as signal functions—administration of parenteral antibiotics, parenteral uterotonics, removal of retained products, manual removal of the placenta, administration of parenteral anticonvulsants, and assisted vaginal delivery. On the other hand, comprehensive EmOC (CEmOC), which is usually only available at higher-level secondary and tertiary facilities, includes all the services available at the basic level plus blood transfusion and surgery (including caesarean section). Prompt access to EmOC can reduce deaths resulting from complications of pregnancy and childbirth amongst women who reach health facilities by as much as 50% (16, 17). However, care for pregnant women requiring EmOC is typically costlier than those with an uncomplicated delivery, so it is apt that the care is covered within robust insurance schemes. However, before a pregnant woman with a complication can access EmOC, she must first travel to a facility that can provide such care. According to Penchansky et al. (18), access to healthcare should incorporate physical availability, geographical accessibility, and affordability. As such, when planning health insurance schemes, it is important to consider geographical accessibility to care in selecting/mainstreaming health facilities to ensure that EmOC is available and accessible to all pregnant women (19). With emerging recognition of the higher capability of global positioning satellite navigation software such as Google Maps to generate closer-to-reality travel time estimates compared to more commonly used modelled approaches such as cost-friction approach and open-source route mapping (20), it will be helpful for informed decision-making and planning for scaling up the health insurance scheme to apply such a tool to access geographical accessibility. We have used this approach to broadly assess geographical accessibility in the most populated Nigerian cities (21) and the linkage between geographical accessibility and poverty (22). However, to the best of our knowledge, we are not aware of any application or use case of such tools to inform the planning of health insurance schemes. This current study aims to assess the geographical accessibility of CEmOC in the context of the Lagos State Health Scheme using spatial analysis, with the utilisation of Google Maps Platform's internal directions API to generate close-to-reality travel times.

## 2 Materials and methods

### 2.1 Study setting

**Figure 1:**
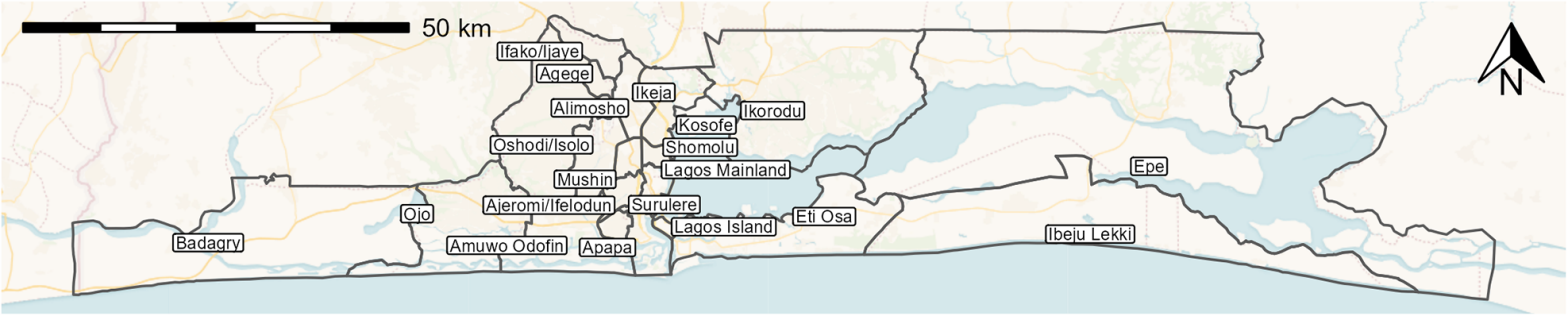
Map of Lagos State with administrative boundaries of local government areas.

Our research examines Lagos, the most populous city in sub-Saharan Africa, with an estimated population of approximately 26 million in 2019, with a density of 6,871 residents per square kilometre (23). Lagos State is highly urbanised and has a range of topographies including a central megacity; suburbs such as Ojo, Ibeju-Lekki, Ikorodu, and Epe surrounding the central megacity; and over 157 slum areas, interspersed all over the state (24). The state is divided into 20 local government areas (LGAs) including a central business district called, Lagos Island (Figure 1).

As per the most recent Nigeria health facility registry, Lagos State has over 2,207 health facilities, of which 81% are public and 19% are private. The health facilities are also spread across the three-tier health system (primary (67% of all), secondary (32%), and tertiary (<1%)) (25). In Lagos specifically, the primary level includes primary healthcare and comprehensive primary health centres (PHCs), secondary includes general hospitals, and tertiary includes tertiary and specialist hospitals. Private facilities in the state include those owned by religious organisations, military, and individuals. Typically, CEmOC is available at secondary and tertiary levels of care and in some private hospitals. A 2022 Health Facility Survey revealed that 26 public and 770 private CEmOC facilities in Lagos State could provide caesarean section at any time of the day (26). Generally, the cost of care is cheaper in the public compared to the private sector (27).

Three comprehensive PHCs, all secondary and tertiary state government-owned (public) health facilities, and 140 private health facilities were registered on the panel of the state's health insurance scheme, bringing to a total of 166 facilities, as of December 2022 (28). There are also two federal government-owned tertiary health facilities that are not listed on the panel. Before a health facility is registered on the panel, the scheme's management agency [Lagos State Health Management Agency (LASHMA)] conducts a visit to the facility to assess its capacity for service provision. Presently, the funds to run the scheme are pooled from the Lagos State Health Fund, which is made up of the premium contributions of members and 1% of the consolidated state's revenue for vulnerable and indigent persons. The annual insurance premium has been set at 8,500 (US$8.5 as of September 2023) per single individual and 40,000 (US$40) for a family of up to six persons. For families larger than six, an additional cost of 6,000 (US$6) is required for every additional family member younger than 18 years and 8,500 (US$8.5) for those older than 18 years (14). Service users are encouraged to use their nearest facility if they need to access care but are not constrained to use facilities within their specific LGA of habitation (14). As of January 2022, over 600,000 persons had been enrolled on the scheme, and in July 2023, LASHMA mandated all private health maintenance organisations to enrol their clients on the state's scheme (14, 29).

### 2.2 Study design

This study combines a health facility assessment, geographic data collection, and secondary data collection of population distribution for women of childbearing age (WoCBA) aged 15–49 years and insurance panel status of health facilities to aid the spatial analysis conducted in this study. The study is part of a broader study that assessed geographical accessibility to EmOC across Nigeria (21, 30).

### 2.3 Data collection

We verified and geocoded functional public and private CEmOC facilities based on their capacity to provide caesarean section and assembled population distribution for women of childbearing age. CEmOC verification, using the capacity to perform caesarean sections as a proxy for all services, was conducted through a facility functionality assessment survey conducted between March and August 2022, using a short questionnaire administered by trained research assistants who paid on-site visits to each facility (26). We subsequently verified the status of health facilities as registered providers of *Ìlera Èkó* using the list of panel providers published by LASHMA in December 2022. After pulling all the data described above, we used the Google Maps Platform's internal directions application programming interface (API) to obtain driving times to public and private facilities (31). This data was extracted in January 2023. The driving times were based on motorised transport from grid centres of approximately 600 m × 600 m (32), as the origin to the nearest CEmOC facility by ownership (public or private). This resolution was selected to balance between accuracy and computation needed for analysis. Details of the methods used to obtain travel time have been published elsewhere (26). Separately, we obtained the population distribution of WoCBA at 1 km^2^ spatial resolutions from the WorldPop open spatial demographic data portal (33).

### 2.4 Data analysis

We aggregated travel time from the grid centres to estimate median travel time (MTT) at higher-level administrative levels including the wider state and sub-state LGA levels. State-level MTT and number of CEmOC facilities reachable within 30 min were calculated for peak hours (during the weekday evening peak at 6–8 p.m.) by the insurance status of facilities. MTT and the number of CEmOC facilities reachable within 30 min at peak hours by the insurance status of facilities were also calculated at the LGA level. We used the population distribution of WoCBA at 1 km^2^ spatial resolutions to compute the LGA-level insurance facility-to-population ratio, reported as the number of facilities per 1,000 WoCBA. We conducted an analysis and visualisation as static maps with R version 4.2.0 (R Development Core Team, Auckland, New Zealand) and ArcMap (ESRI ArcGIS, Redlands, CA, USA). The data used for the analysis are publicly available and described in detail elsewhere (26).

## 3 Results

### 3.1 Number of CEmOC facilities in total and those included in the insurance scheme, per LGA

**Figure 2:**
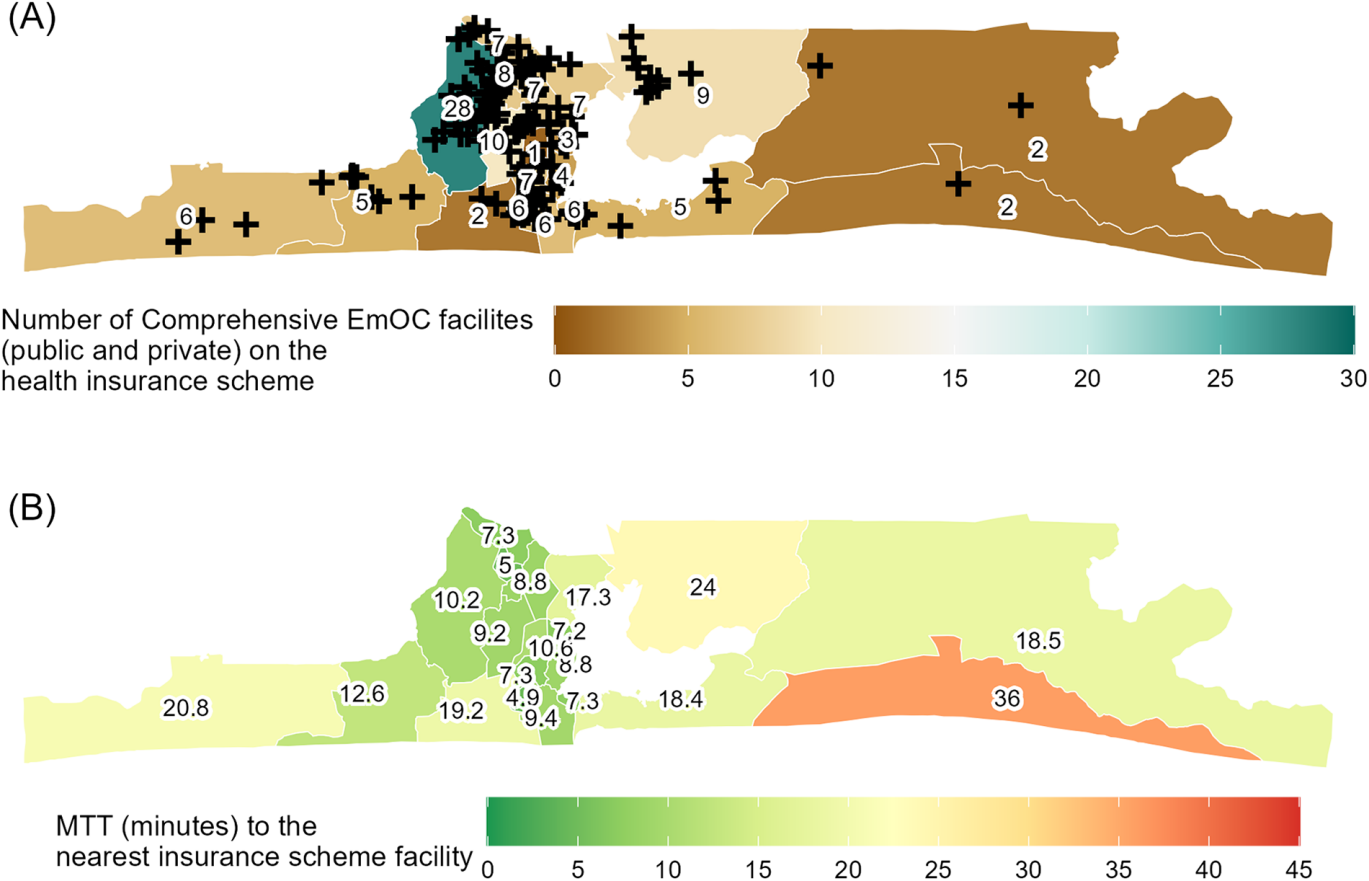
Choropleth maps of Lagos State local government areas showing **(A)** CEmOC facilities (both public and private) on the health insurance scheme per 1,000 WoCBA and **(B)** median travel time (MTT) in minutes to the nearest insurance scheme facility.

Of the 796 facilities assessed to be able to provide CEmOC in Lagos based on the facility functionality assessment survey, 26 are public and 770 are private. From this list, 23 out of the 26 (88%) public and 108 out of the 770 (14%) private facilities were on the panel of the social health insurance scheme in the state, as of December 2022. The number of CEmOC facilities on the panel per LGA ranged from 1 in Mushin LGA to 28 in Alimosho (Figure 2A).

### 3.2 Geographical accessibility in the context of the insurance scheme

In Lagos, overall, MTT to the nearest public CEmOC was 25 min. Private facilities that were part of the insurance scheme and could provide CEmOC helped to expand coverage and reduce travel time to affordable care to 17 min (public or private). MTT to the nearest public facility in the LGAs were between 9 min in Lagos Island and 51 min in Ojo (median = 18 min). Other LGAs with relatively long MTT were Ibeju-Lekki (41 min), Badagry (35 min), and Ikorodu (31 min). MTT to either public or private CEmOC facilities on the insurance panel across LGAs was found to be between 5 min in Agege and 36 min in Ibeju-Lekki (median = 13 min). The largest reduction in MTT when public or private facilities were considered was observed in Ojo LGA (51–13 min). Ibeju-Lekki remained with MTT above 30 min (36 min) with public and private facilities considered while Mushin (11 min), Ojo (13 min), Kosofe (17 min), Eti-Osa (18 min), Amuwo-Odofin (19 min), Epe (19 min) Badagry (21 min), and Ikorodu (24 min) had an MTT exceeding 10 min but under 30 min with public and private insurance scheme facilities considered (Figure 2B; Table 1).

**Table 1:** Median travel time (MTT) to the nearest CEmOC facility.

	MTT to the nearest CEmOC facility				Average number of CEmOC facilities reachable within 30 min of driving			
	Public facility^a^	Public or panelled private facilities on insurance panel	Difference between public or panelled private facilities and public only	Panelled private facilities only	Public facility^a^	Public or panelled private facilities on insurance panel	Difference between public or panelled private facilities and public only	Panelled private facilities only
Ojo	51	13	38	13	0	4	4	4
Ibeju-Lekki	41	36	5	82	0	0	0	0
Badagry	35	21	14	22	0	2	2	1
Ikorodu	31	24	7	26	0	3	3	2
Amuwo-Odofin	30	19	11	20	1	8	7	6
Alimosho	25	10	15	11	0	13	13	12
Eti-Osa	25	18	6	24	0	4	4	2
Kosofe	21	17	4	18	5	18	13	14
Ifako/Ijaye	19	7	12	8	1	15	14	14
Epe	19	19	1	99	1	1	0	0
Oshodi-Isolo	17	9	8	11	3	16	13	12
Ikeja	16	9	7	10	4	25	21	21
Agege	13	5	8	5	1	34	33	32
Surulere	13	7	6	8	8	25	17	19
Apapa	13	9	3	10	5	20	15	15
Ajeromi-Ifelodun	12	5	7	5	5	21	16	17
Mushin	11	11	0	11	10	24	14	17
Shomolu	11	7	3	9	9	23	14	17
Lagos Mainland	10	9	1	11	8	23	15	16
Lagos Island	9	7	1	7	4	16	12	12
*City-wide median*	25	17		22	1	7		6

^a^ Public facility includes all public facilities on the state insurance scheme and the two federally owned tertiary public facilities.

On average, the number of public CEmOC facilities that were reachable within 30 min of driving for women living in each of the LGAs was ≤5 in 16 of 20 LGAs, including 0 in Alimosho, Eti-Osa, Ojo, Ikorodu, Badagry, and Ibeju-Lekki. In five of these six LGAs, the additional consideration of private CEmOC facilities within the insurance scheme led the number of facilities reachable within 30 min of driving for women living in each of the LGAs increased to 13, 4, 4, 3, and 2 (Alimosho, Eti-Osa, Ojo, Ikorodu, and Badagry, respectively), whilst that in Ibeju-Lekki remained 0 (Table 1).

## 4 Discussion

### 4.1 Summary and interpretation of results

We set out to use the Google Maps Platform's internal directions API to generate closer-to-reality travel time estimates to explore the geographical accessibility to CEmOC within the context of a social health insurance scheme in Lagos, Nigeria. We found that the number of CEmOC facilities varied by LGA, with more public or private CEmOC facilities on the insurance panel situated in the central more urban parts of the state and fewer in the less urban extremities. The number of public CEmOC facilities is spread evenly among the LGAs (21); however, this is not the case for the panelled private CEmOC facilities. The higher number of CEmOC facilities in the central areas might be because the area has more people living there and as such attractive to private EmOC service providers as opposed to the peripheral areas with fewer persons.

Across the entire state, MTT to the nearest public CEmOC (insurance or not) was 25 min and reduced to 17 min when public and private facilities on the insurance panel were considered. A previous study reported that 62% of pregnant women in Lagos arrived at a public CEmOC facility where they received care within 30 min driving time (34). Nonetheless, MTT to public or private facilities for WoCBA living in various LGAs varied, with those in slum areas such as Mushin, Ojo, and Kosofe and suburbs such as Eti-Osa, Amuwo-Odofin, Epe, Badagry, and Ikorodu having MTT greater than 10 min to reach affordable CEmOC in the state. This travel time benchmark is particularly significant as a previous study that assessed the influence of travel time on the actual public CEmOC where care was received on stillbirth showed that the odds of stillbirth more than doubled with 10 min driving time in Lagos (35). Notably, the most significant decrease in MTT with the inclusion of private facilities on the insurance panel was observed in Ojo LGA, with MTT reducing by 38 min. Conversely, in areas such as Epe, Mushin, Lagos Island, and Mainland, the inclusion of private facilities led to little or no change in MTT.

On average, no public CEmOC facility within the health insurance scheme was reachable within 30 min driving in Badagry, Alimosho, Eti-Osa, Ibeju-Lekki, Ikorodu, and Ojo LGAs. These are all suburbs in Lagos, many of which were classed as ‘hotspots’ requiring longer travel to care in a previous Lagos study, which used travel data of pregnant women who presented in an emergency to public CEmOC facilities (36). With facilities from the insurance panel added however, the average number of CEmOC facilities reachable within 30 min driving increased in certain LGAs with the most increase seen in Ikeja, the capital city (21), and Agege, its adjoining suburb (33). Ibeju-Lekki remained with zero facilities within 30 min on average. Given disparities in geographical accessibility, policymakers could prioritise the inclusion of more private CEmOC facilities in the LGAs with fewer CEmOC facilities within the 30 min travel time threshold, especially when scaling the LASHMA scheme. This approach would play a pivotal role in bridging the significant gaps in accessibility.

### 4.2 Strengths and limitations

Regarding strengths, first, this study represents an inaugural attempt at employing closer-to-reality travel time estimation to evaluate geographical accessibility to CEmOC within the context of a policy aimed at addressing financial accessibility (20, 37). Moreover, only health facilities that have been verified to have the capacity to provide CEmOC were included. However, there are limitations. First, pregnant women do not always travel to the nearest facility for care, even in an emergency (38). However, for this scheme, pregnant women are encouraged to use their nearest facility for care and would most likely do so to ensure they can benefit from the scheme (14, 38). Furthermore, the basis of UHC hinges on the provision of care to a certain minimum standard both locally and nationally. Second, we took the pragmatic decision to consider caesarean section capacity as a proxy for CEmOC services. However, it is also likely that there will be some facilities that can provide caesarean sections but are not able to provide some of the other EmOC services. Third, the insurance panel database and travel time data used were captured in December 2022 and January 2023, respectively. As such, our analysis does not reflect the new private facilities that have been since included (15).

### 4.3 Implications for policy and practice

Despite the limitations, there are highly relevant implications for policy that have emerged from the study. Put together, our findings strengthen the justification of incorporating private CEmOC facilities in the health insurance scheme, as they not only minimise the risk of catastrophic expenditure for pregnant women but have also reduced MTT to affordable CEmOC across the state. However, the reduction may not yet be clinically significant to make a difference in terms of pregnancy outcomes, which is the long-term goal of the scheme (13). This is even more critical in the suburbs and slum areas, where MTT remained over 10 min for WoCBA, which means they remain with higher odds for death following complications of pregnancy and childbirth and delivering stillbirths (34, 35). As such, while we cannot rule out potential improvements in geographical accessibility to facilities on the panel that might have serendipitously occurred since the addition of new facilities, the government needs to adopt a more strategic approach in facility selection that targets these areas of geographical inequities to identify capable private facilities that can be mainstreamed into the panel list of facilities to bring affordable care closer to women and save lives. Nonetheless, solely increasing the onboarding of private facilities should not substitute the development of more public facilities, especially for a suburb like Ibeju-Lekki where there are no facilities accessible within 30 min and MTT difference with the addition of private facilities on the panel made only minimal difference of 5 min in MTT (41–36 min). There are certainly communities like this which are far away from the more cosmopolitan areas of the state that are less attractive for private sector providers and even more so high-quality ones (39). Furthermore, some women in Lagos prefer public hospitals for delivery due to the perception of a wider range of specialist services available (40).

The social health insurance scheme in Lagos expanded the number of facilities that can provide affordable CEmOC reachable within 30 min and reduced MTT to such facilities broadly, although disparities remain across LGAs. Stakeholders involved in planning the scheme can draw on the findings of this study in prioritising private facility selection in LGAs with higher MTT. Although patterns of service utilisation and provision may differ across cities (41), the innovative approach of using closer-to-reality travel time estimates linked with data from a registry of verified health facilities that are part of the insurance scheme offers policy-relevant evidence to support efforts of African governments in advancing UHC and ensuring coverage for a critical service such as EmOC to those who are most vulnerable.

## 5 Conclusion

Social health insurance schemes such as the Lagos State Health Scheme presented in this brief research report are strategic policy instruments that help to minimise the risk of exposure to catastrophic health exposure that pregnant women and their families may face in emergency situations. The cost of such services can be significantly higher in crisis situations (42, 43). The findings from this study show that in designing and implementing social health insurance schemes, geographical accessibility to panelled facilities needs to be considered if failure of such schemes is to be averted and their goal of realising UHC realised (44).

## Data Availability

The datasets presented in this study can be found in online repositories. The names of the repository/repositories and accession number(s) can be found below: https://doi.org/10.6084/m9.figshare.22699759.v1.
